# Lipid core nanoparticles resembling low-density lipoprotein and regression of atherosclerotic lesions: effects of particle size

**DOI:** 10.1590/1414-431X20177090

**Published:** 2018-03-01

**Authors:** S.C.M.P. Freitas, E.R. Tavares, B.M.O. Silva, B.C. Meneghini, R. Kalil-Filho, R.C. Maranhão

**Affiliations:** 1 Instituto do Coração, Faculdade de Medicina, Universidade de São Paulo, São Paulo, SP, Brasil; 2 Faculdade de Ciências Farmacêuticas, Universidade de São Paulo, São Paulo, SP, Brasil

**Keywords:** Solid lipid nanoparticles, Drug targeting, Paclitaxel, Atherosclerosis, Particle size

## Abstract

Particles are usually polydispersed and size is an important feature for lipid-based drug delivery systems in order to optimize cell-particle interactions as to pharmacologic action and toxicity. Lipid nanoparticles (LDE) with composition similar to that of low-density lipoprotein carrying paclitaxel were shown to markedly reduce atherosclerosis lesions induced in rabbits by cholesterol feeding. The aim of this study was to test whether two LDE fractions, one with small (20–60 nm) and the other with large (60–100 nm) particles, had different actions on the atherosclerotic lesions. The two LDE-paclitaxel fractions, prepared by microfluidization, were separated by density gradient ultracentrifugation and injected (4 mg/body weight, intravenously once a week) into two groups of rabbits previously fed cholesterol for 4 weeks. A group of cholesterol-fed animals injected with saline solution was used as control to assess lesion reduction with treatment. After the treatment period, the animals were euthanized for analysis. After treatment, both the small and large nanoparticle preparations of LDE-paclitaxel had equally strong anti-atherosclerosis action. Both reduced lesion extension in the aorta by roughly 50%, decreased the intima width by 75% and the macrophage presence in the intima by 50%. The two preparations also showed similar toxicity profile. In conclusion, within the 20–100 nm range, size is apparently not an important feature regarding the LDE nanoparticle system and perhaps other solid lipid-based systems.

## Introduction

Among the several physical characteristics of solid nanoparticles designed for drug delivery, the particle size is a prominent one (1,2). Size can be determinant for the passage of nanoparticles through physiologic barriers, such as normal vessel walls, blood-brain barrier, blood-ocular barrier or the fenestrations of tumor neovasculature (3,4). In addition, regarding the nanoparticles that can actively bind to targeted cells, the affinity of the ligands with cell membrane surfaces can also be largely dependent of particle size (5). Size also determines the stereochemical relations of the ligand to the cell binding elements, such as receptors or other surface proteins or non-protein compounds (6).

In previous studies, we have shown that cholesterol-rich nanoparticles termed LDE are taken-up by the low-density lipoprotein (LDL) receptors after injection into the bloodstream (7). Nanoparticles are made to mimic the lipid composition of LDL, having a phospholipid monolayer, small amounts of cholesterol surrounding a core of cholesteryl esters, and residual triglycerides (8). Although LDE do not contain protein, the LDL-like nanoparticles acquire apo E and other apolipoproteins from native lipoproteins in contact with plasma. Apo E is recognized by and allows binding of the LDL-like nanoparticles to the receptors (9–16). As cancer cells show upregulation of LDL receptors, LDE can be used as vehicle to direct antineoplastic drugs to those cells. Indeed, it was shown in *in vitro* studies that LDE internalizes drugs such as carmustine, etoposide, and paclitaxel into cultured neoplastic cells (7,17,18 [Bibr B19] [Bibr B20] [Bibr B21]). Injected into patients with cancer, LDE concentrated in breast and ovary carcinoma tissues, acute myelocytic leukemia, and multiple myeloma (14,). In studies enrolling patients with advanced multi-drug resistant cancers, it was shown that carmustine, etoposide, and paclitaxel showed a remarkable reduction of clinical and laboratorial toxicity when associated to LDE (12,17,23).

Subsequently, LDE was shown also to concentrate in non-neoplastic cell lines under rapid proliferation, such as erythroid precursors in beta-thalassemia patients and in atherosclerotic lesions of cholesterol-fed rabbits (3,11). These findings prompted us to plan pioneering studies on the introduction of anti-cancer drugs in atherosclerosis therapeutics. This hypothesis was based on the anti-proliferative and immunosuppressive actions of the drugs that can be effective in reducing lesions and atherosclerosis-related inflammation. Indeed, drugs such as paclitaxel, etoposide, methotrexate, and carmustine associated to LDE had the ability to markedly reduce the lesions and the concurrent proliferative and inflammatory processes (24).

Nanoparticles are defined by the International Organization for Standardization as a material having dimensions between 1 and 100 nm (25,26). In this study, we hypothesized whether the nanoparticle size of this lipid-based drug-delivery system could influence the therapeutic action of paclitaxel associated to nanoparticles on atherosclerotic lesions induced in rabbits. LDE system particles stand at the 20–100 nm range, with average 50–70 nm diameter by laser light scattering. If size is influential in the therapeutic action of associated drugs, technical efforts could be made to select the size range of those preparations that bear considerable polydispersity. Clarification of this issue would be important not only for this specific artificial lipoprotein system, but also to general lipid-based systems and other drug-delivery strategies.

## Material and Methods

### Preparation of LDE associated to paclitaxel oleate

The LDE-paclitaxel oleate preparation was made according to the methods described in previous studies (11,27,28). Briefly, a lipid mixture composed by 135 mg cholesteryl oleate (Alfa Aesar, USA), 333 mg egg phosphatidylcholine (Lipoid, Germany), 132 mg miglyol 812N (Sasol Germany GmbH, Germany), 6 mg cholesterol (Fabrichem, USA) and 60 mg of paclitaxel oleate (Pharmaceuticals Co., China), was added to an aqueous phase consisting of 100 mg of polysorbate 80 (Merck, Germany) and 10 mL Tris-HCl buffer, pH 8.05. A pre-emulsion was obtained by ultrasonic radiation until complete solubilization of the drug. Emulsification of all lipids, drug and aqueous phase was obtained by high-pressure homogenization using an Emulsiflex C5 homogenizer (Avestin, Canada). After homogenization cycles, the formed emulsion was centrifuged and the nanoparticle sterilized by passage through 0.22 μm pore polycarbonate filter (Millipore, USA) and kept at 4°C until used.

### Separation of LDE into two particle size ranges

LDE paclitaxel has produced in polydisperse lipid particles. For selection of various particle diameters, we used a technique similar to that used for the separation of lipoproteins by density gradient (29). To measure the density of all solutions before and after adjustments to salt potassium bromide (KBr), a digital densitometer was used (Mettler Toledo International Inc., USA).

The nanoparticle density was adjusted to 1.21 g/mL with KBr and 6 mL of LDE-paclitaxel were placed on 12.5 cm ultracentrifugation tubes (polyallomer tubes, Beckman, USA). To form the first gradient, 2 mL of 0.001 M TRIS-HCl with a density of 1.045 g/mL were slowly added to each of these tubes. To this solution, 2 mL of 0.001 M TRIS-HCl, density 1.020 g/mL, were added to form the second gradient. Tubes containing the preparations were ultracentrifuged (Ultracentrifuge Optima XL 100K swing rotor SW 41 Ti, Beckman) at 247,163 *g* for 20 h at 4°C.

At the end of the ultracentrifugation process, four fractions were visible, separated by density gradient. The top fraction (density 1.020 mg/mL) concentrated particles of diameter between 70–90 nm, while the third fraction (density 1.045) concentrated particles of 30–50 nm of average diameter. Then, each fraction was aspirated and packaged in dialysis membranes for 24 h in 2 L of TRIS-HCl 0.001 M solution for KBr removal. After dialysis, the fractions were sterilized again by a polycarbonate filter with 0.22 µm pore size.

The average diameter, polydispersity index and zeta potential of the two fractions were determined by dynamic light scattering method at a 90° angle, using the Zetasizer Nano ZS90 equipment (Malvern, UK). Under unidirectional laminar airflow, 100 µL samples of each fraction were diluted in 1 mL of TRIS-HCl 0.1 M buffer, and placed in polyacrylamide cuvettes to perform particle diameter and zeta potential measurements. The upper major fraction will now be called large LDE-paclitaxel, with a diameter 70–100 nm, and the lower fraction will be called small LDE-paclitaxel, with a diameter of 30–50 nm. These two fractions were used in all experiments described below.

The efficiency of the association of paclitaxel oleate to the original large and small nanoparticles was analyzed by high-performance liquid chromatography (HPLC; Shimadzu, USA) method in isocratic mode, mobile phase 90% methanol and 10% acetonitrile with an UV-visible detector at 234 nm. The final concentration of paclitaxel oleate associated with LDE was calculated using the calibration curve.

### Animals and experimental protocol

This project was approved by the Ethics Committee for Research Project Analysis (CAPPesq-2939/07/014) of the Instituto do Coração (InCor), and by the Ethics Committee in Use of Animals (CEUA 050/14), of the Hospital das Clínicas, Faculdade de Medicina (FM), Universidade de São Paulo (USP), São Paulo, SP, Brazil.

Male New Zealand white rabbits weighing 3.0 to 3.5 kg were used. The animals were kept under controlled temperature and light cycle of 12 h. Water was provided *ad libitum*. Rabbits received 150 g/day of a cholesterol-rich diet for 8 weeks. The remaining portion was weighed daily to evaluate the amount of chow consumed by the animals during the study. Animals were weighed weekly.

After 4 weeks, rabbits were allocated in three groups: control group (n=9): animals received intravenous injections of saline solution once a week, for 4 weeks; large-LDE-paclitaxel group (n=9): animals received intravenous injections of large LDE-paclitaxel (4 mg/kg) once a week for 4 weeks; small LDE-paclitaxel (n=10): animals received intravenous injections of LDE-small paclitaxel (4 mg/kg) once a week for 4 weeks.

### Laboratory tests

Blood samples of the rabbits were collected from the lateral ear vein before the beginning of the cholesterol-rich diet, before the beginning of the treatment with LDE-paclitaxel and at the end of the study for determination of total and high density cholesterol (HDL), triglycerides, alanine aminotransferase (ALT), aspartate aminotransferase (AST), urea, creatinine, and for blood cell count. The analyses were performed using a COBAS c111 (Roche, Switzerland) and a veterinary hematology analyzer Poch 100iV Diff Sysmex-Roche (Roche) at the Special Analysis Laboratory (LAR) of the Hospital das Clínicas, FM, USP.

### Macroscopic analysis of atherosclerotic lesions

Animals were euthanized with a lethal dose of thiopental (Tiopentax, Brazil) at the end of the protocol. The aorta was excised from the aortic arch to the abdominal artery, then opened longitudinally and fixed in 10% formalin. The lipid deposits in the aorta were stained by Scarlat R (Sudan IV; Sigma, USA), and aortas were photographed to perform the measurements. Total area and lesions area were measured using Leica QWin Image Analysis software (Leica Q500 iW; Leica Imaging Systems, UK) and the percentage of macroscopic atherosclerotic lesions was calculated by the ratio lesion area/total area.

### Morphometric analysis of histological sections of the aortic arch

After completion of the macroscopic examination, the region of the aortic arch of the arteries were sliced into fragments of 5 mm, embedded in paraffin and cut into 5-μm sections. The slides were deparaffinized and stained with hematoxylin-eosin. Total and intima layer area were measured, and the percentage of lesion was calculated by the ratio intima area/total area.

### Immunohistochemistry

Additional sections were labeled with anti-clone RAM11 antibody for rabbit macrophages (Dako, USA). For immunostaining, antigen retrieval was performed with a Pascal antigen retrieval high-pressure chamber (Dako, Denmark) with 10 mM citrate buffer, pH 6.0. Endogenous peroxidase activity was blocked by incubation in 3% hydrogen peroxide, and nonspecific reaction was blocked by incubation in 1% bovine albumin (Sigma-Aldrich, USA) for 1 h at 37°C. The sections were then incubated overnight at 4°C with anti-RAM11. Next, the sections were incubated for 30 min at room temperature with the Envision Polymer Detection System (Dako). The sections were then incubated with a 3,3′-diamino-benzidine (DAB) chromogen system (Dako) for 2 min at room temperature and counterstained with hematoxylin. The image analysis of immunostaining for macrophages was calculated by the percentage of labeled area relative to total tissue area. The color detection threshold was chosen for the DAB chromogen (brown staining) in tissue sections. The measurements were performed using the QWin Image Analysis software (Leica Imaging Systems).

### Statistical analysis

Data are reported as means±SE. Data were analyzed using one-way ANOVA complemented by Bonferroni's post-test, or Kruskal-Wallis with Dunn's post-test. In all analyses, P<0.05 was considered statistically significant. Statistical analyses were carried out using GraphPad Prism v.7 statistical software (GraphPad Software, Inc., USA).

## Results

### Separation of LDE-paclitaxel according to size

The small particle fraction had an average diameter of 40.69 nm±1.44, polydispersity index of 0.043±0.01 and pH 7.95±0.1. The average diameter of the large particle fraction was 83.61±1.85 nm, with a polydispersity index of 0.130±0.04 and pH 7.94±0.1. The average paclitaxel concentration of the 8 analyzed samples in the small particle fraction was 5.70±0.35 mg/mL (95%CI=5.1–6.2 mg/mL) and the large particle fraction was 7.27±1.10 mg/mL (95%CI=6.4–8.1 mg/mL; P<0.01). Zeta potential of the large particle fraction was 8.4±0.7 mV and the small particle fraction was 6.5±0.7 mV.

### Food intake and body weight before and after treatment

**Figure 1.**
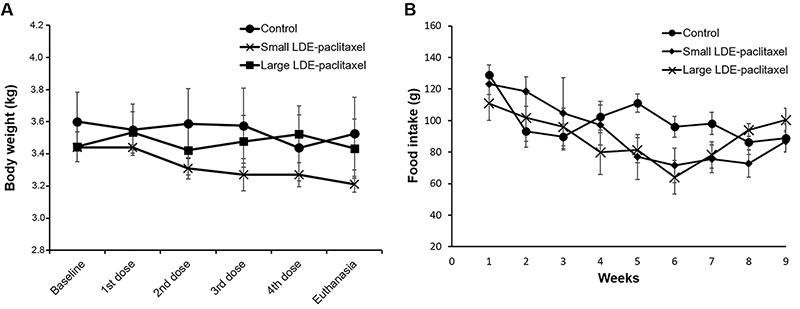
Body weight and food intake. *A*, Body weight (kg) of small lipid nanoparticles (LDE)-paclitaxel and large LDE-paclitaxel groups. *B*, Food intake (g) of the same groups. Data are reported as means±SE, using one-way ANOVA with Bonferroni's post-test.

As shown in Figure 1, there was no significant difference in food intake among the control group and small and large particles LDE-paclitaxel treated groups. The evolution of body weight was similar in control and in both LDE-paclitaxel treated groups.

### Plasma lipids

Table 1 shows the plasma lipid profile of the rabbits at baseline and at 4 and 8 weeks after the beginning of 2-month cholesterol-rich dietary period for the three groups. As expected, after the introduction of the daily high cholesterol intake, the values of total lipids raised roughly 20-fold and HDL cholesterol about 10-fold. The triglyceride values also pronouncedly increased.

**Table 1.** Biochemical analysis of rabbits from control group (n=9) and treated with small lipid nanoparticles (LDE)-paclitaxel (n=10) and large LDE-paclitaxel (n=9).

	Control			Small LDE-paclitaxel			Large LDE-paclitaxel		
	Baseline	Pre-treatment	Post-treatment	Baseline	Pre-treatment	Post-treatment	Baseline	Pre-treatment	Post-treatment
Cholesterol (mg/dL)									
Total	42±4	1172±63^a^	1528±96^a^,^d^	44±7^b^	1266±262^a^,^d^,^f^	1467±236^a^,^d^	55±14^c^,^e^	1064±184^d^	1058±144^d^
HDL	22±2	259±23	311±70	14±3^b^,^c^	197±11	318±27^a^,^d^,^f^	16±4^b^	210±30^d^,^f^	270±37^a^,^d^,^f^
Non-HDL	20±3	913±70^a^	1217±99^a^	30±7^b^,^c^	1069±255^a^,^d^,^f^	1148±220^a^,^d^,^f^	39±12^b^,^c^	854±99^a^,^d^,^f^	787±116^a^,^d^,^f^
Triglycerides (mg/dL)	66±9	153±40	211±41	88±13	94±18	231±54	83±8	74±13	196±56
ALT (U/L)	59±12	66±11^f^	65±7^d^,^f^	35±4^a^	55±8	50±11	26±3^a^	51±9	63±10
AST (U/L)	34±5	56±15	61±11	30±5	64±9	63±10	37±5	51±9	63±13
Urea (mg/dL)	33.5±2.4	44.8±4.7^f^	49.3±5.5	27.9±1.4^c^	38.4±7.9	45.2±3.6^d^,^f^	26.3±1.8^c^	37.0±4.7	48.5±2.0^d^,^f^
Creatinine (mg/dL)	0.97±0.06	1.43±0.16^d^,^f^	1.62±0.20^d^	0.58±0.05	0.88±0.14^c^	1.18±0.06^d^,^f^	0.50±0.08	1.00±0.21	1.19±0.09^d^,^f^

Data are reported as means±SE. HDL: high density cholesterol; ALT: alanine aminotransferase; AST: aspartate aminotransferase.

a P<0.05 *vs* Control baseline;

b P<0.05 *vs* Control pre-treatment;

c P<0.05 *vs* Control post-treatment;

d P<0.05 *vs* Small LDE-paclitaxel baseline;

e P<0.05 *vs* Small LDE-paclitaxel post-treatment;

f P<0.05 Large LDE-paclitaxel baseline (ANOVA with Tukey's post-test).

### Toxicity evaluation

Table 1 also shows the data on liver enzymes, urea and creatinine. ALT and AST values were similar in all three groups. Urea and creatinine increased in control and in both treated groups.

Both groups treated with LDE-paclitaxel particles showed decreased values of red blood cells, hemoglobin concentration and hematocrit percentage compared to control animals (Table 2).

**Table 2.** Hematological profile of rabbits from control group (n=9) and treated with small lipid nanoparticles (LDE)-paclitaxel (n=10) and large LDE-paclitaxel (n=9).

	Control			Small LDE-paclitaxel			Large LDE-paclitaxel		
	Baseline	Pre-treatment	Post-treatment	Baseline	Pre-treatment	Post-treatment	Baseline	Pre-treatment	Post-treatment
Erythrogram									
Erythrocytes (10^9^/mL)	5.9±0.5	4.4±0.2	3.4±0.3	5.6±0.2	4.1±0.4	2.8±0.2^a^,^b^	6.0±0.3^c^	3.4±0.5^b^,^c^	3.3±0.2^a^,^b^,^c^
Hemoglobin (g/dL)	12.4±0.6	10.3±0.4	9.4±0.5	12.0±0.4	9.6±0.7	7.4±0.4^a^,^b^,^c^	12.5±0.6	8.3±0.9^c^	8.4±0.5^a^,^b^,^c^
Hematocrit (%)	38±1	31±1^b^,^c^,^d^	29±2^a^,^b^,^c^,^d^	38±1	29±2^a^,^b^,^c^,^d^	21±1^a^,^b^,^c^	39±2	27±2^a^,^b^,^c^	24±1^a^,^b^,^c^
Leukogram									
Leucocytes (10^6^/mL)	11.0±1.2	16.7±1.1^b^	22.5±2.2^a^,^b^,^c^	9.4±0.8	15.2±1.8	14.6±1.5	11.0±0.8	12.0±1.3	12.5±1.3
Neutrophils (%)	42±4	36±2	31±4	39±4	32±4	43±3	39±4	25±3	35±3
Lymphocytes (%)	55±3	58±2	61±4	58±4	65±5	54±3	57±4	71±3	61±3
Monocytes (%)	3±0.5	3±0.4	5±0.4	4±0.5	3±0.3	3±0.4	4±0.5	4±0.5	4±0.7
Platelets (10^3^/mm^3^)	246±26	286±39	263±44	194±20	262±24	223±25	188±20	192±30	200±38

Data are reported as means±SE.

a P<0.05 *vs* Control baseline;

b P<0.05 *vs* Small LDE-paclitaxel baseline;

c P<0.05 *vs* Large LDE-paclitaxel baseline;

d P<0.01 *vs* Small LDE-paclitaxel post-treatment (ANOVA with Tukey's post-test).

Also in Table 2, total leukocyte count in the control group increased during the dietary period (P<0.01), but no difference was observed in both groups treated with LDE-paclitaxel. Neutrophil, lymphocyte, and monocyte counts, as well as the platelet count were unaltered by treatment in both groups of animals.

### Evaluation of the atherosclerotic lesions

**Figure 2.**
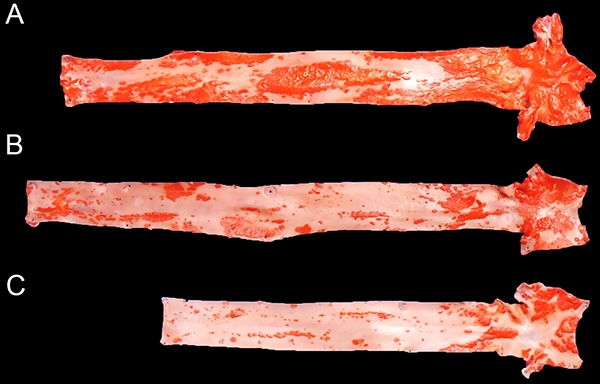
Atherosclerotic lesions on rabbit aortas. *A*, control group, *B*, small lipid nanoparticles (LDE)-paclitaxel group, and *C*, large LDE-paclitaxel group stained by Scarlat R (Sudan IV).

Figure 2 shows pictures of the aorta of rabbits treated with saline solution, small LDE-paclitaxel and large LDE-paclitaxel. As expected, the aortic arch was the segment exhibiting the densest area of atheromatous lesions compared with the thoracic and abdominal segments. After treatment, both sizes of LDE-paclitaxel had equally strong anti-atherosclerosis action (P<0.01 *vs* control; Table 3).

**Table 3.** Macroscopic and microscopic morphometry of rabbit aortas treated with saline solution (n=9), small lipid nanoparticles (LDE)-paclitaxel (n=10) or large LDE-paclitaxel (n=9).

	Control	Small LDE-paclitaxel	Large LDE-paclitaxel
Macroscopy			
Total area (pixel^2^ ×10^6^)	0.68±0.69	1.32±0.41*	1.28±0.12*
Lesion area (pixel^2^ ×10^6^)	0.46±0.53	0.41±0.39*	0.36±0.32*
% of lesion	64.3±4.8	29.7±7.7*	28.1±8.2*
Microscopy			
Total area (µm^2^ ×10^6^)	4.33±3.86	1.12±0.26*	1.05±0.17*
Lesion area (µm^2^ ×10^6^)	1.82±1.40	0.11±0.13*	0.10±0.10*
% of lesion	39.0±7.6	9.5±3.0*	8.7±2.6*
% Macrophages in the intima	66.4±4.1	31.3±7.8*	31.5±5.2*

Data are reported as means±SE.

* P<0.01 *vs* control (ANOVA with Tukey's post-test).

**Figure 3.**
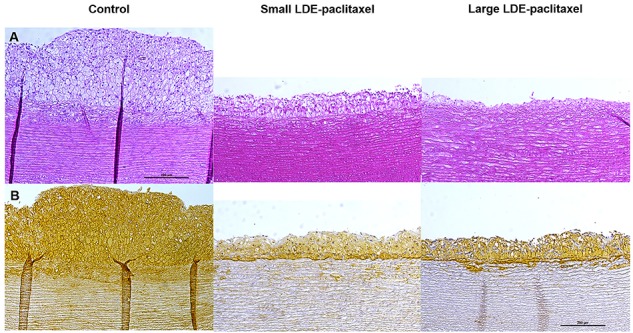
*A*, Representative photomicrographs of aortic arch stained by hematoxylin-eosin of control, small lipid nanoparticles (LDE)-paclitaxel and large LDE-paclitaxel groups. *B*, Immunohistochemistry of macrophage stained area on artery tissues from the three groups. Brown staining indicates macrophages stained by DAB chromogen. Magnification: 100×. Bar: 200 µm.

The photomicrographs of Figure 3 show segments of the aortic arch of rabbits treated with saline solution, small LDE-paclitaxel and large LDE-paclitaxel, stained with hematoxylin-eosin and anti-rabbit macrophages. Animals treated with the two fractions of LDE-paclitaxel showed a smaller percentage of lesion (P<0.001 *vs* control) and a diminished presence of macrophages in the intima layer (P<0.01 *vs* control; Table 3). Both treatments reduced lesion extension in the aorta by roughly 50%, decreased the intima width by 75% and the macrophage presence by 50%.

## Discussion

In this study, it was shown that the size of the lipid nanoparticles, at least within the 20–100 nm diameter range, was not determinant for treatment outcome of paclitaxel carried in LDE in rabbits with atherosclerosis.

In regards to the LDE system, the size of the nanoparticles is presumptively of chief importance for their cell uptake (30,31). LDE nanoparticles bind to LDL receptors and possibly to lipoprotein-related protein receptors (22). As LDL receptors take-up LDL with size ranging from 20–30 nm, it would be expected that the small particle fraction of LDE-paclitaxel would be taken-up by the LDL receptors more avidly than the large particle fraction (32–34). This would result in greater influx of paclitaxel into the cytoplasm of the cells involved in atherogenesis and greater anti-atherosclerotic action (35). However, LDL receptors also take-up much larger lipoprotein particles than LDL, such as intermediate density lipoproteins (IDL) and chylomicron remnants, which are taken-up by those receptors using apo E as ligand. LDL binds to those receptors through apo B, which is the only lipoprotein present on LDL particle surface. Apo E has much more affinity for LDL receptors than apo B (around 20- to 30-fold), so that chylomicron remnants and IDL are removed from the circulation much faster than LDL (36). In this context, our finding that small and large fractions have equivalent ability to promote atherosclerosis regression is not unexpected. Nevertheless, nanoparticles with a diameter much above 100 nm would not be eligible to be taken-up by LDL receptors, but rather more prone to be phagocytized by macrophages. Indeed, it is established that larger particles are trapped by macrophage scavenger receptors (37–39). The effects of LDE-paclitaxel on the atherosclerotic lesions for the two preparations, small and large nanoparticles, were not much different from those found for LDE-paclitaxel documented in our previous study (11). In that study, 60% lesions reduction were found in the cholesterol-fed rabbits, with similar reductions in intima area and presence of macrophages.

In view of our data, the nanoparticle size in this lipid-based drug targeting system is apparently not important when tailoring the nanoparticles for large scale production. This finding is important not only for the LDE system but also for other lipid core nanoparticle systems that depend on membrane receptors for internalization into cells. If substantial differences were found for preparations with different particles sizes, the need for a technical effort to achieve particles with smaller or larger sizes would be mandatory, aiming to optimize the pharmacological action of the nanocarrier system. This endeavor would not be simple, since changes in composition and physicochemical features required for size reduction or increase might impair the functionality of the nanoparticles. The concern on this issue is greatly dispersed by our results as they clarify an important issue for future trials testing the actions of LDE-paclitaxel in patients with atherosclerotic cardiovascular disease.
